# Abnormal neuroinflammation in fibromyalgia and CRPS using [^11^C]-(R)-PK11195 PET

**DOI:** 10.1371/journal.pone.0246152

**Published:** 2021-02-08

**Authors:** Seongho Seo, Ye-Ha Jung, Dasom Lee, Won Joon Lee, Joon Hwan Jang, Jae-Yeon Lee, Soo-Hee Choi, Jee Youn Moon, Jae Sung Lee, Gi Jeong Cheon, Do-Hyung Kang

**Affiliations:** 1 Department of Electronic Engineering, Pai Chai University, Daejeon, Republic of Korea; 2 Department of Psychiatry, Seoul National University Hospital, Seoul, Republic of Korea; 3 Department of Psychiatry, Kangdong Sacred Heart Hospital, Seoul, Republic of Korea; 4 Department of Medicine, Seoul National University College of Medicine, Seoul, Republic of Korea; 5 Department of Psychiatry, Seoul National University College of Medicine and Institute of Human Behavioral Medicine, SNU-MRC, Seoul, Republic of Korea; 6 Department of Anesthesiology and Pain Medicine, Seoul National University Hospital, Seoul, Republic of Korea; 7 Department of Biomedical Sciences, Seoul National University College of Medicine, Seoul, Republic of Korea; 8 Institute of Radiation Medicine, Medical Research Center, Seoul National University, Seoul, Republic of Korea; 9 Department of Nuclear Medicine, Seoul National University College of Medicine, Seoul, Republic of Korea; 10 Emotional Information and Communication Technology Association, Seoul, Republic of Korea; University of Modena and Reggio Emilia, ITALY

## Abstract

**Purpose:**

Fibromyalgia (FM) and complex regional pain syndrome (CRPS) share many pathological mechanisms related to chronic pain and neuroinflammation, which may contribute to the multifactorial pathological mechanisms in both FM and CRPS. The aim of this study was to assess neuroinflammation in FM patients compared with that in patients with CRPS and healthy controls.

**Methods:**

Neuroinflammation was measured as the distribution volume ratio (DVR) of [^11^C]-(R)-PK11195 positron emission tomography (PET) in 12 FM patients, 11 patients with CRPS and 15 healthy controls.

**Results:**

Neuroinflammation in FM patients was significantly higher in the left pre (primary motor cortex) and post (primary somatosensory cortex) central gyri (p < 0.001), right postcentral gyrus (p < 0.005), left superior parietal and superior frontal gyri (p < 0.005), left precuneus (p < 0.01), and left medial frontal gyrus (p = 0.036) compared with healthy controls. Furthermore, the DVR of [^11^C]-(R)-PK11195 in FM patients demonstrated decreased neuroinflammation in the medulla (p < 0.005), left superior temporal gyrus (p < 0.005), and left amygdala (p = 0.020) compared with healthy controls.

**Conclusions:**

To the authors’ knowledge, this report is the first to describe abnormal neuroinflammation levels in the brains of FM patients compared with that in patients with CRPS using [^11^C]-(R)-PK11195 PET. The results suggested that abnormal neuroinflammation can be an important pathological factor in FM. In addition, the identification of common and different critical regions related to abnormal neuroinflammation in FM, compared with patients with CRPS and healthy controls, may contribute to improved diagnosis and the development of effective medical treatment for patients with FM.

## Introduction

Fibromyalgia (FM) is a complex condition characterized by chronic widespread pain and multiple other symptoms, including fatigue, sleep disturbances, cognitive dysfunction, memory problems, stiffness, and comorbid anxiety and depression [1, 2]. Although FM has historically defied explanation based on peripheral theories of pain, individuals with these disorders exhibit significant alterations in central nervous system (CNS) factors that lead to augmented pain and sensory processing [3]. FM is considered a syndrome of abnormal central pain processing and increased central sensitivity caused by neurobiological changes that cause dysregulation of mechanisms that normally regulate pain sensation [4]. Complex regional pain syndrome type I (CRPS I), formerly known as reflex sympathetic dystrophy (RSD), is a chronic painful disorder that usually develops after any known trauma or other lesion [5]. Possible pathophysiological mechanisms of CRPS I are that an increased input from peripheral nociceptors alters the central processing mechanisms such as neurogenic inflammation, and maladaptive neuroplasticity [5].

[^11^C]-(R)-PK11195 is a ligand of positron emission tomography (PET) for a translocator protein (TSPO) that is expressed by activated microglia or astrocytes [6]. Evidence of neuroinflammation is supported by the activated microglia or astrocytes, which demonstrate an increased expression of the TSPO [6]. We used [^11^C]-(R)-PK11195 and PET to investigate the existence of neuroinflammation in FM patients. An enhanced inflammatory state and inflammatory-stress feedback dysregulation underlies FM [7]. Chronic pain is maintained, in part, by central sensitization, and increased neuronal responsiveness in central pain pathways after painful insults, and central sensitization is also driven by neuroinflammation in the peripheral nervous system and CNS [8]. Neuroinflammation is associated with the activation of glial cells, including microglia and astrocytes, in the brain, leading to the release of pro-inflammatory cytokines and chemokines, not only in the pathogenesis of various neurodegenerative disorders, but also in chronic and neuropathic pain [8, 9]. Neuroinflammation, which is characterized by activation of glial cells and the production of inflammatory mediators in the peripheral nervous system and CNS, plays an important role in the induction and maintenance of chronic pain, suggesting that addressing excessive neuroinflammation could reveal new therapeutic targets for treating chronic pain [10]. Additionally, it has been proposed that inflammation could be a mitochondrial dysfunction-dependent event implicated in the pathophysiology of FM, suggesting mitochondria as a possible new therapeutic target [11]. Although neuroinflammation has been found as a pathology in FM, as assessed in cerebrospinal fluid (CSF) [12] and PET [13], assessing neuroinflammation in the brains of FM patients using PET need to be more explored.

Several brain structures involved in pain processing and pain modulation have been investigated using neuroimaging in attempts to understand abnormal pain sensitivity [14]. FM is correlated with decreases in gray matter volume in specific brain regions, decreased functional connectivity in the descending pain-modulating system, and increased activity in the pain matrix related to central sensitization [15]. Abnormally increased pain-related brain activity has been identified within the thalamus, insula, anterior cingulate, S1, and prefrontal cortex (the so-called ’pain matrix’) of patients with FM [16].

The aim of this study was to assess neuroinflammation using [^11^C]-(R)-PK11195-PET in FM patients compared with that in patients with CRPS and healthy controls. Although we reported increased neuroinflammation in patients with CRPS using [^11^C]-(R)-PK11195 PET in our previous study [17], we reanalyzed neuroinflammation levels in CRPS patients using the same analyses as those of FM patients to compare neuroinflammation levels and to identify distinct features between these patient groups. Finally, we investigated the relationship between neuroinflammation levels and psychiatric symptoms in FM patients.

## Methods

### Participants

The present study included 12 patients who fulfilled the American College of Rheumatology criteria for FM [18], and were recruited from the National University Hospital. In addition, 11 CRPS patients who fulfilled the International Association for the Study of Pain criteria for CRPS (Budapest criteria) and had been also recruited in the authors’ previous study [17] were included. Finally, 15 individuals, who were of comparable age and gender with the FM and CRPS patients, and exhibited no pain or neurological symptoms, were used as healthy controls. The subjects used as the healthy controls were the same used in a previous CRPS study [17]. 12 patients with FM and additional control subjects were recruited using Internet advertisements for 7 months. The number of study participants was based on a sample size calculation for a comparison of distribution volume ratio (DVR) between groups. Effect size was estimated from available PK11195 pilot study [19]. In addition, we used G*power 3.1.9.2 (Heinrich Heine, Universität Düsseldorf, Germany) program [20] with unpaired t test, an 80% power while controlling type I error rate at 5%. The computed sample size was 11 subjects for each group, so we planned to recruit 15 subjects for each group considering potential withdrawal of subjects.

Subjects who exhibited high levels of high-sensitivity C-reactive protein (hs-CRP) or leukocytosis were excluded. The inclusion criteria for FM subjects were as follows: diagnosis of FM; age between 21 and 63 years; and not taking benzodiazepine or could discontinue benzodiazepine medication 2 weeks before the study. Individuals with a major neuropsychiatric disorder before the diagnosis of FM, a neurological disease (cerebrovascular disease or brain tumor), a history of brain trauma, high levels of hs-CRP or leukocytosis, as well as those who could not undergo the PET/magnetic resonance imaging (MRI) were excluded. Sensory and affective dimensions of current pain were assessed using the McGill pain Questionnaire Short-Form (SF-MPQ), which contains 11 McGill Pain Questionnaire-Sensory (MPQ-S) and 4 McGill Pain Questionnaire-Affective (MPQ-A) pain items [21]. Stress levels in the FM patients were assessed using the Stress Response Inventory (SRI), which consists of 39 items (score range: 0–156) categorized into 7 factors: fatigue, tension, frustration, anger, depression, somatization, and aggression [22]. The post-traumatic stress disorder (PTSD) Check List (PCL) assesses the development of PTSD in respondents after exposure to a traumatic event [23]. This study was approved by the Institutional Review Board (IRB) at the Seoul National University Hospital (IRB No. 1703-138-841). All data were obtained under written informed consent granted by all subjects after a full explanation of the experimental methods.

### Synthesis of [^11^C]-(R)-PK11195

[^11^C]-(R)-PK11195 was synthesized using a previously reported method with a few modifications [24]. The precursor and the cold-standard of [^11^C]-(R)-PK11195 were purchased from ABX (Radeberg, Germany); the other chemicals were obtained from Sigma-Aldrich Korea (Kyunggi-do, Korea). Briefly, (R)-N-desmethyl-PK11195 (the precursor of [^11^C]-(R)-PK11195, 1 mg, 2.8 μmol) and sodium hydride (60% in mineral oil, 7 mg, 486 μmol) were dissolved in 500 μL of dimethyl sulfoxide in a reaction vial. [^11^C]CH_3_I was bubbled into the reaction vial for 10 minutes, and the reaction mixture was allowed to proceed for an additional 5 minutes. The reaction mixture was purified by preparative high-performance liquid chromatography (HPLC) (Xterra C18 column, 10 × 250 mm^2^, 10 μm, Waters Corp.; Milford, MA) with the eluent (water:ethanol  =  55:45, 5 mL/min). The final product solution was reconstituted with 10 mL of a saline solution. The purity of the final product was checked by analytical HPLC (YMC-Triart C18 column, 4.6 × 100 mm^2^, 3 μm, YMC Co., Ltd.; Kyoto, Japan) with the eluent (water:MeCN  =  40:60, 1 mL/min). The radioactivity of [^11^C]-(R)-PK11195 was 5.7 ± 1.8 GBq (156.7 ± 51.3 mCi [n  =  10]); the specific activity was 215.2 ± 93.7 GBq/μmol (5.8 ± 2.5 Ci/mol [n  =  10]). The total synthesis time, including formation and purification, was less than 40 minutes from the end of bombardment.

### PET/MR image acquisition

Each participant underwent a 60 min dynamic [^11^C]-(R)-PK11195 PET scan using a PET/MRI scanner (Biograph mMR, Siemens Healthcare GmbH, Erlangen, Germany). PET was performed using a spatial resolution of 4.4 mm at 1 cm and 5.2 mm full-width at half-maximum (FWHM), with a 10 cm offset from the center of the transverse field of view. The details were described in the authors’ previous study [17].

### Quantification of binding

A parametric image of the DVR for each participant was generated using relative equilibrium-based graphical analysis with a reference-region input [25]. For the reference region, the region of interest (ROI) of the cerebellum was delineated on a simultaneously acquired sagittal T1-weighted MR image. For the extraction of ROI mean values and subsequent statistical analyses, the individual DVR images were spatially normalized by registering the MR images to the MNI152 template using statistical parametric mapping (SPM) (SPM8; www.fil.ion.ucl.ac.uk/spm). The ROI DVR values were then extracted from the normalized DVR images using population-based probability maps [26, 27], the details of which are described in the authors’ previous study [17].

### Statistical analysis

Statistical analyses of ROI data were performed using SPSS version 21.0 (IBM Corporation, Armonk, NY, USA). The differences in DVR of [^11^C]-(R)-PK11195 in ROI between FM and healthy controls was assessed using analysis of covariance (ANCOVA) controlling for any effects of gender. The differences in DVR in ROI between patients with CRPS and healthy controls were assessed using ANCOVA controlling for the duration effect. The differences in DVR of [^11^C]-(R)-PK11195 in ROI between patients with FM and CRPS were assessed using a two-tailed Student’s t-test. If normality was not satisfied, Mann–Whitney U test was used. Pearson’s correlation analysis was used to evaluate the association between psychological test scores and the DVR of [^11^C]-(R)-PK11195. Differences with P < 0.05 were considered to be statistically significant in the ROI analysis.

To assess group differences at the voxel level, statistical inferences were further performed on the spatially normalized DVR images using SPM8 after 10 mm Gaussian smoothing to reduce the image registration error. ANCOVA was applied for a comparison between FM and healthy controls with gender as a covariate, and between CRPS and healthy controls with duration as a covariate. A two-sample t test was used to compare patients with CRPS and FM. The resulting maps were thresholded at the P = 0.01 level, without correction for multiple comparisons.

## Results

### Study participants and basic information

Twelve FM, 11 CRPS patients, and 15 healthy control subjects completed all study procedures. Demographic and clinical characteristics of the subjects were summarized in Table 1. Age, gender, and education level were not significantly different among the groups. We exhibited CNS medication information for patients’ therapies in S1 Table. FM patients were taking various CNS medications, including opioids (N = 4), anticonvulsants (N = 10), antidepressants (N = 8), antipsychotics (N = 6), anxiolytics (N = 4), antimigraine agent (N = 3), antiparkinson agent (N = 2) and benzodiazepine (N = 1). CRPS patients were also taking CNS medications, including opioids (N = 7), anticonvulsants (N = 7), antidepressants (N = 7), anxiolytics (N = 1), and benzodiazepine (N = 6).

**Table 1:** Participant`s demographic characteristics.

	FM patients	CRPS patients	Healthy controls	*p*-value
N	12	11	15	
Age	41.7±14.0	40.9±8.8	41.3±6.6	***p* = 0.929**, *p* = 0.907
Gender	5M, 7F	8M, 3F	10M, 5F	***p =* 0.182,** *p* = 0.543
Education(years)	14.4±2.6	14.8±2.4	16.2±2.0	***p* = 0.054**, *p* = 0.120
Duration of illness(years)	4.4 ± 3.1	8.9 ± 5.3	N/A	

Data are mean ± standard deviation. **Bold *p*** shows p-value between FM and healthy controls. Non-bold *p* shows p-value between CRPS and healthy controls. **FM**: Fibromyalgia, **CRPS**: Complex Regional Pain Syndrome; **M** = male; **F** = female.

### Differences in neuroinflammation between FM and healthy controls

**Fig 1:**
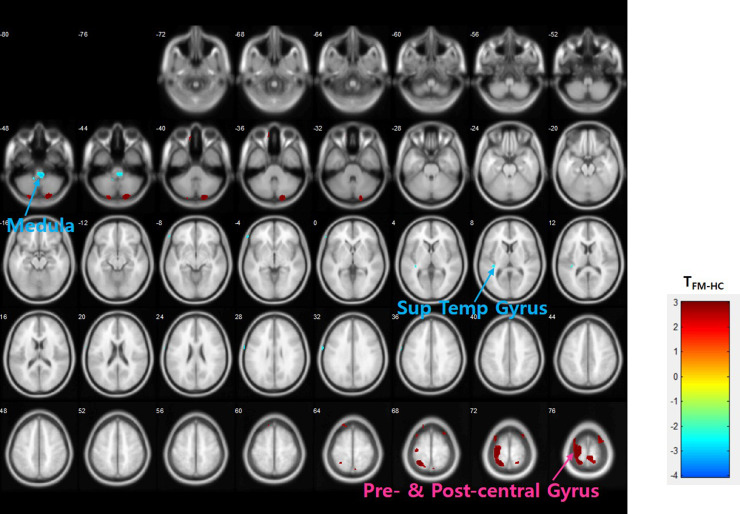
Differences in neuroinflammation indexed as DVR of [^11^C]-(R)-PK11195 between FM and healthy controls. Analysis of covariance (ANCOVA) with gender covariate, uncorrected p < 0.01.

The voxel-wise ANCOVA, controlled for the effect of gender, revealed significant differences in neuroinflammation (i.e., DVR of [^11^C]-(R)-PK11195) between the FM and healthy control group. The FM group exhibited decreased [^11^C]-(R)-PK11195 binding in the medulla (p < 0.005) and the left superior temporal gyrus (p < 0.005) compared with the healthy control group (Fig 1), but an increased [^11^C]-(R)-PK11195 DVR in the left pre- and post-central gyri (p < 0.001), the right postcentral gyrus (p < 0.005), the left superior parietal and superior frontal gyri (p < 0.005), and the left precuneus (p < 0.01) (Fig 1). Additionally, ROI analysis controlling for the effect of gender revealed lower binding in the medulla (p = 0.001) and left amygdala (p = 0.020), but higher binding in left medial frontal (p = 0.036) in FM patients compared with that in healthy controls.

### Differences in neuroinflammation between CRPS patients and healthy controls

**Fig 2:**
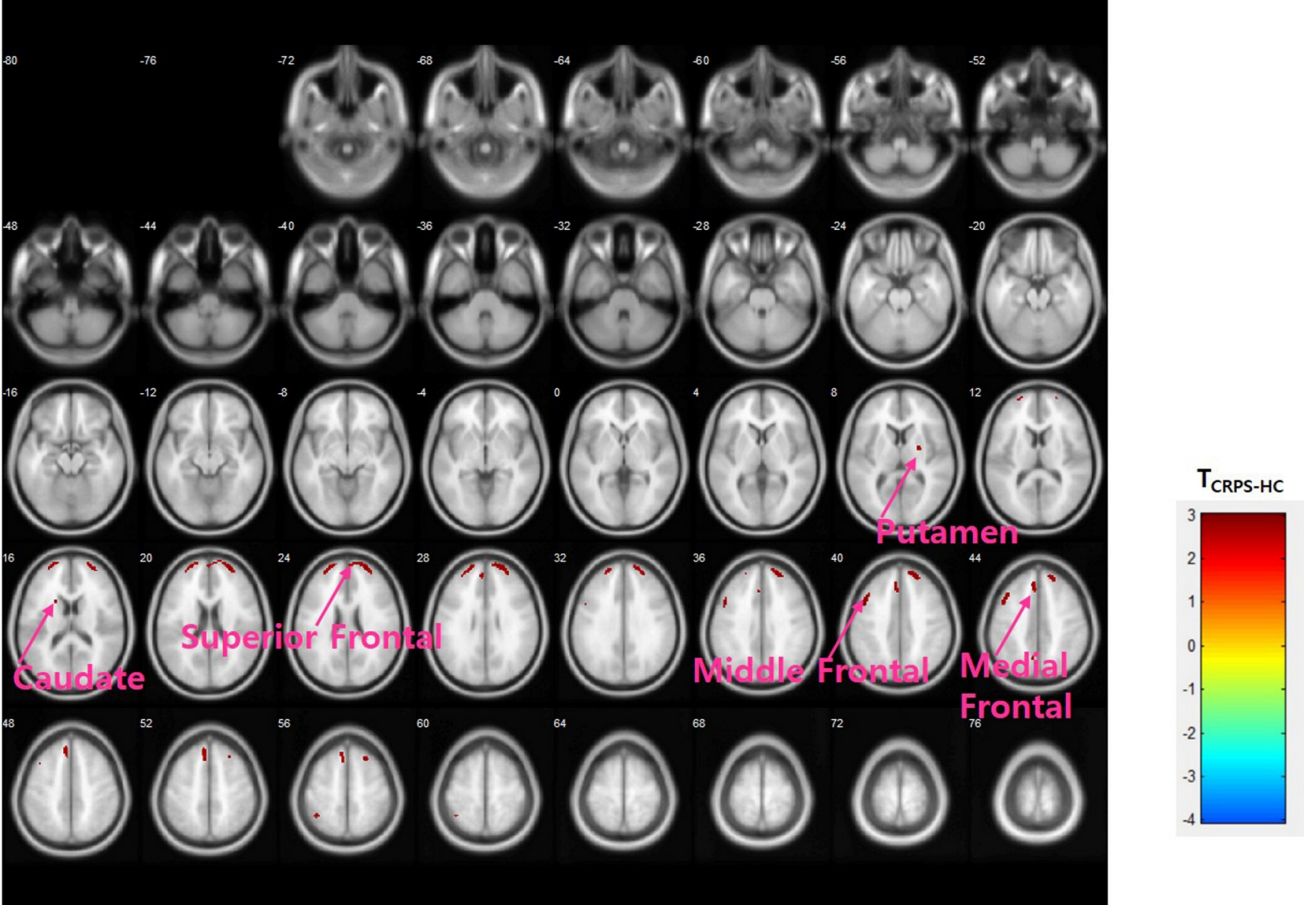
Differences in [^11^C]-(R)-PK11195 DVR between CRPS patients and healthy controls. Analysis of covariance (ANCOVA) with duration covariate, uncorrected p < 0.01.

When accounting for the duration effect in comparing the CRPS and healthy control groups, abnormally higher [^11^C]-(R)-PK11195 binding levels in the CRPS group was detected in the left superior, middle and medial frontal gyri (p < 0.01, p < 0.01, and p < 0.001, respectively), the left inferior and superior parietal gyri, the left precuneus (p < 0.01), the left caudate (p < 0.01), the right putamen (p < 0.01), and the right superior, middle, and medial frontal gyri (p < 0.005) (Fig 2). Additionally, using ROI analysis while controlling for the duration effect in the comparison of CRPS and healthy control groups, a higher [^11^C]-(R)-PK11195 DVR was found in the in right insula (p = 0.033), left thalamus (p = 0.042), left precentral gyrus (p = 0.027), right and left postcentral (p = 0.018, p = 0.025, respectively), right and left middle frontal (p = 0.001, p = 0.004), right and left medial frontal (p = 0.009, p = 0.006), right and left superior frontal (p = 0.011, p = 0.010), left lateral orbitofrontal (p = 0.029), left superior parietal (p = 0.035), right and left caudate head (p = 0.014, p = 0.013), right and left nucleus accumbens (p = 0.007, p = 0.020), left putaman (p = 0.004), left caudate (p = 0.019), left globus pallidus (p = 0.050), and right superior marginal (p = 0.012) in CRPS patients.

### Differences in neuroinflammation between FM and CRPS patients

**Fig 3:**
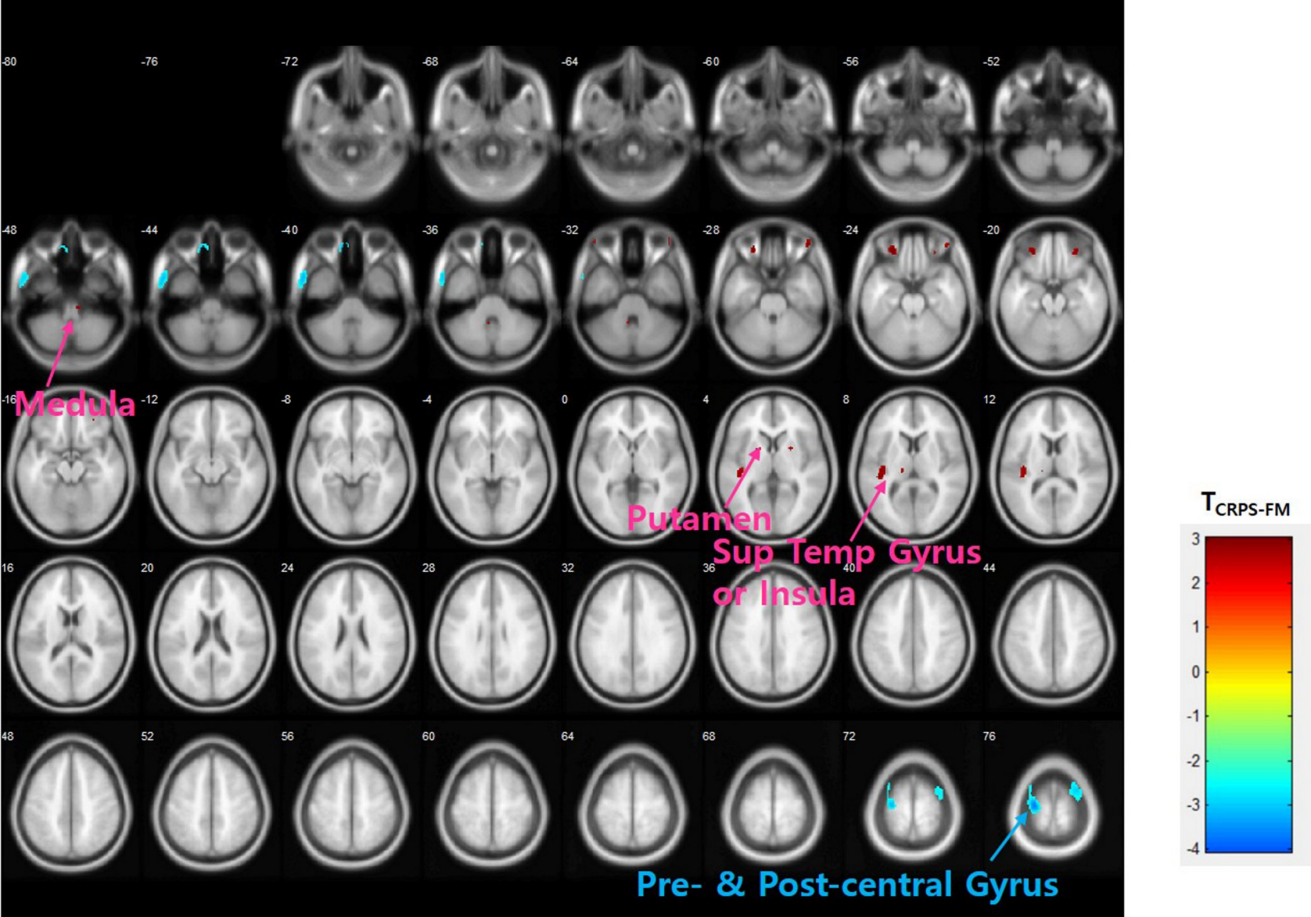
Differences in DVR of [^11^C]-(R)-PK11195 between FM and CRPS patients. Two sample t-test, uncorrected p < 0.01.

Higher neuroinflammation levels in the left pre- and post-central gyri were found in the FM group compared with the CRPS group (p < 0.001 and p < 0.005, respectively) (Fig 3). On the other hand, higher binding levels in the CRPS group were observed in the medulla, left insula, left thalamus, left superior temporal gyrus, the right and left putamen, and the right and left medial orbital gyri compared to that of the FM group (p < 0.01) (Fig 3). Additionally, ROI analysis revealed higher [^11^C]-(R)-PK11195 binding in the right and left thalamus (p = 0.023, p = 0.008) right and left putamen (p = 0.008, p = 0.046), right and left caudate (p = 0.012, p = 0.029), right and left caudate head (p = 0.005, p = 0.034) and right globus pallidus (p = 0.037) of CRPS patients compared with that of the FM group.

### Correlations between neuroinflammation and psychological symptoms in FM patients

**Fig 4:**
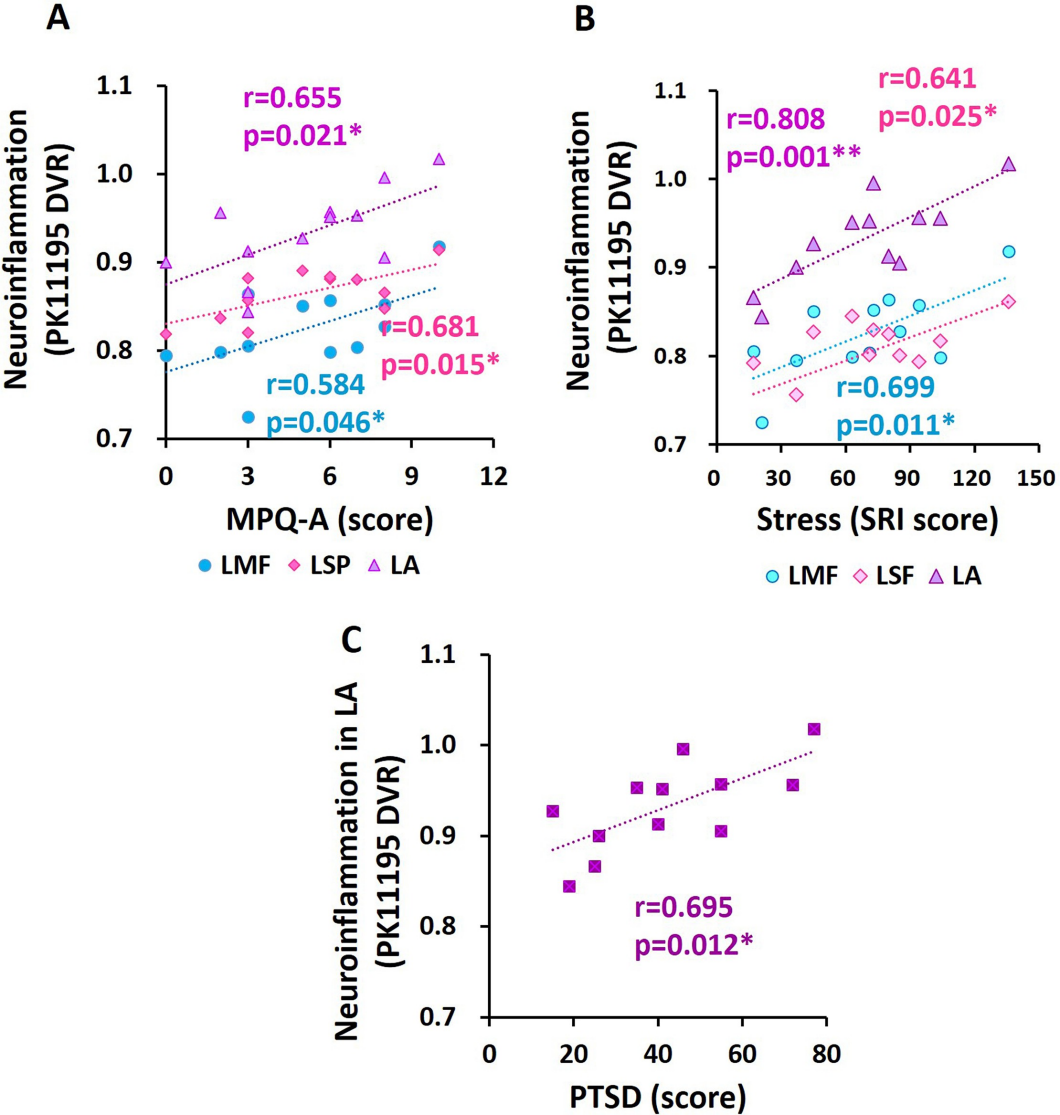
Correlations between affective pain, stress and PTSD and neuroinflammation ([^11^C]-(R)-PK11195 DVR) in FM. **(A-C)** LMF: left medial frontal, LSF: left superior frontal, LSP: left superior parietal, LA: left amygdala. MPQ-A: McGill pain Questionnaire-Affective, SRI: Stress Response Inventory, PTSD: posttraumatic stress disorder. *p < 0.05, ** p < 0.01.

In patients with FM, there were positive correlations between the DVR of [^11^C]-(R)-PK11195 in the left medial frontal (r = 0.584, p = 0.046), left superior parietal (r = 0.681, p = 0.015) and left amygdala (r = 0.655, p = 0.021) and the affective pain score (MPQ-A) (Fig 4A). In patients with FM, there were positive correlations between the DVR of [^11^C]-(R)-PK11195 in the left medial frontal (r = 0.699, p = 0.011), left superior frontal (r = 0.641, p = 0.025) and left amygdala (r = 0.808, p = 0.001) and SRI stress scores (Fig 4B). Furthermore, the DVR of [^11^C]-(R)-PK11195 in the left amygdala demonstrated a positive correlation with PTSD scores (r = 0.695, p = 0.012) in FM patients (Fig 4C).

## Discussion

**Fig 5:**
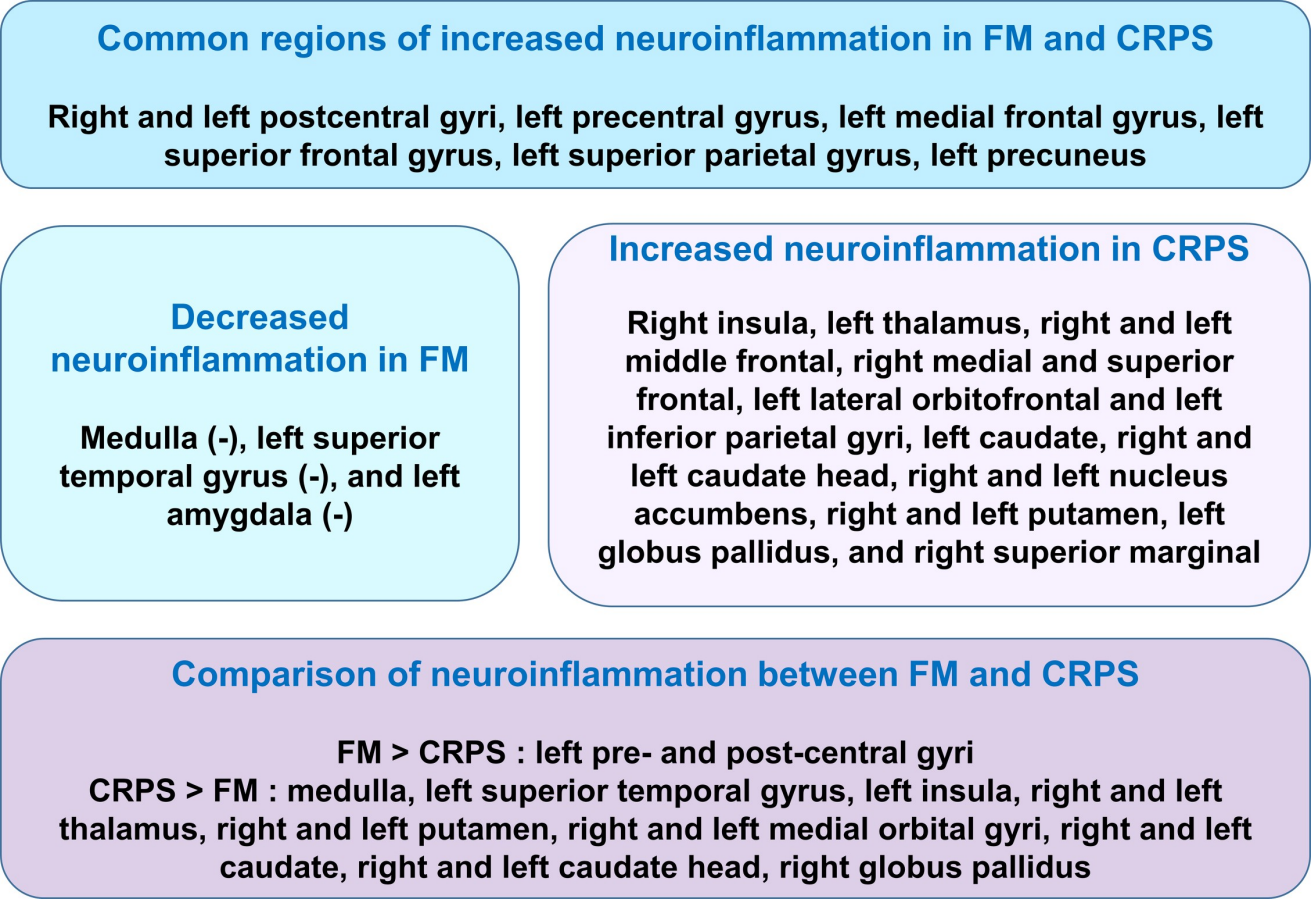
Common and different regions of abnormal neuroinflammation in FM and CRPS patients. **FM**: fibromyalgia, **CRPS**: complex regional pain syndrome. **(-)**: Decreased neuroinflammation.

In the present study, we found abnormal neuroinflammation levels in brain using [^11^C]-(R)-PK11195 PET in FM patients. Recent study reported widespread cortical elevations of neuroinflammation, as assessed using the [^11^C]PBR28 PET ligand which binds to the TSPO, demonstrating microglia, but not astrocytes, may be driving the TSPO elevation in FM patients [13]. The TSPO elevations in M1, S1, precuneus and superior parietal lobe in the recent study [13] were in part coincide with our results, whereas the study showed no decreases in TSPO binding in FM patients. However, this study also found the decrease of neuroinflammation in some brain regions such as medulla, left amygdala and left superior frontal gyrus in FM patients. In addition, this study demonstrated abnormal neuroinflammation levels in the brains of FM patients compared with that in patients with CRPS using [^11^C]-(R)-PK11195 PET. Thus, our results may exhibit more extensive and detailed findings related to abnormal neuroinflammation in FM patients. In addition, we found common and different abnormal neuroinflammation levels in some brain regions in FM compared with patients with CRPS. For example, we identified common abnormally higher neuroinflammation levels in the right and left postcentral gyrus (primary somatosensory cortex), left precentral (primary motor cortex), left medial frontal, left superior frontal, left superior parietal gyri, and left precuneus in FM and CRPS patients (Fig 5), implying that higher neuroinflammation in these regions would be associated with neuronal pathological mechanisms of chronic pain of FM and CRPS. In particular, the precentral gyrus is the site of the primary motor cortex, and the postcentral gyrus is the location of the primary somatosensory cortex, the main sensory receptive area for the sense of touch. The somatosensory cortex is important for the cortical representation of pain [28] and to the perception of noxious stimuli [29]. Therefore, increases in neuroinflammation in the primary somatosensory cortex may contribute to central sensitization from the stimulation of nociceptors by excessive neuroinflammation [8, 30]. On the other hand, stimulation of the rat primary motor cortex (M1) induced spinal antinociception in neuropathic as well as control conditions [31], implying that the primary motor cortex may effect regulation in the descending pain pathway. Thus, dysfunction and high neuroinflammation in the primary motor cortex may contribute to dysregulation in the descending pain pathway and failure in descending modulation of pain [32–34]. Therefore, excessive neuroinflammation in both the primary somatosensory cortex and primary motor cortex may contribute to central sensitization and neuroinflammation from stimulating nociceptors and dysfunction in regulation and modulation of pain in FM and CRPS patients. In addition to the pre- and postcentral gyrus, the frontal cortex is known to control descending inhibition of nociception in cognitive pain modulation [35–38]. Thus, higher neuroinflammation in the left frontal regions appear to contribute to the neuropathology and cognitive dysfunction in both FM and CRPS patients. And, higher neuroinflammation was found in the left precuneus of both FM and CRPS patients in this study. Cortical thinning and decreased connectivity have been found in the left precuneus of PTSD after trauma [39, 40]. Considering FM and CRPS can both be triggered by specific traumatic events [41], abnormal neuroinflammation in left precuneus may be induced by trauma which had been experienced in both FM and CRPS patients.

Although FM and CRPS may share many pathological mechanisms related to chronic pain, we identified different brain regions exhibiting higher or lower neuroinflammation in FM that were distinct from those in CRPS patients (Fig 5). The primary finding in this study was that FM patients exhibited higher neuroinflammation in the pre-central and postcentral gyrus than did CRPS patients and healthy controls. Abnormal pain sensitivity and decreased pain modulation in both the ascending and descending pain systems, including the pre- and postcentral gyrus, may contribute to the main neuropathology of FM [14, 42, 43], which may be associated with neuroinflammation in these regions in FM. On the other hand, we found lower neuroinflammation in some brain regions including the medulla, left amygdala, and left superior temporal gyrus in patients with FM. Lower cortical thickness has been described in the left amygdala, right superior temporal gyrus, right and left middle temporal gyrus, left superior frontal gyrus, and right fusiform gyrus in FM patients compared with healthy controls [44], in addition to decreases in gray matter volume in the prefrontal cortex, the amygdala, and the anterior cingulate cortex (ACC) [45]. Lower cortical thickness and decreased gray matter volume in these regions may be associated with neuronal cell death-related dysregulation of inhibitory pain mechanisms and lower neuroinflammation in FM patients. Lower neuroinflammation in the medulla which is the descending pain control system may be related to descending facilitation in the chronic pain state [46].

In the correlation analysis results, we confirmed an association between higher affective pain levels and higher neuroinflammation in the left medial frontal, left superior parietal and left amygdala in patients with FM. Considering that higher levels of stress and PTSD were related to higher neuroinflammation in FM, psychological trauma and stress may be primary causes that induce and drive the progression of neuroinflammation and pathological symptoms in FM.

This study had a few limitations, the first of which was that it only examined a small number of FM and CRPS type I patients. Thus, future studies will require larger sample sizes to generalize our findings to the broader population of FM and CRPS patients.

In conclusion, the present study identified specific brain regions that exhibit abnormal neuroinflammation in FM using [^11^C]-(R)-PK11195 PET. In addition, we demonstrated common and different regions presenting abnormal neuroinflammation between FM and CRPS patients compared with healthy controls. Based on these results, we propose that the neuropathological mechanisms in FM and CRPS are somewhat different. Our results suggest that abnormal neuroinflammation can be an important pathological factor in FM patients as well as those with CRPS. Furthermore, distinct brain regions where abnormal neuroinflammation is exhibited in FM can provide important clues related to the cause of disease in FM patients and distinguish them from those with CRPS, which, moreover, may play an important role in developing effective medical treatments for patients with FM and CRPS.

## Supporting information

S1 Table CNS medication for patients’ therapies. (DOCX)

S1 Data (SAV)
