# Supplementary material for: Abnormal neuroinflammation in fibromyalgia and CRPS using [11C]-(R)-PK11195 PET
Source: PLoS One. 2021 Feb 8;16(2):e0246152. doi: 10.1371/journal.pone.0246152 (PMC7870009; doi:10.1371/journal.pone.0246152)
Supplement: S1 Table — (DOCX) [file pone.0246152.s001.docx]

**S1 Table.** **CNS medication for patients’ therapies**

| **Patients** | **CNS medication** |
| --- | --- |
| FM 01 | anticonvulsant, antidepressant, antiparkinson agent |
| FM 02 | anticonvulsant, antidepressant, anxiolytics, antimigraine agent |
| FM 03 | opioid, anticonvulsant, antipsychotics, anxiolytics |
| FM 04 | opioid, antipsychotics, antidepressant |
| FM 05 | anticonvulsant, antipsychotics, antidepressant |
| FM 06 | opioid, anticonvulsant, antidepressant |
| FM 07 | anticonvulsant, antidepressant |
| FM 08 | anticonvulsant, antipsychotics, anxiolytics |
| FM 09 | anticonvulsant, antipsychotics, antidepressant, anxiolytics, antimigraine agent |
| FM 10 | anticonvulsant, antidepressant, benzodiazepine, antiparkinson agent |
| FM 11 | No medication |
| FM 12 | opioid, anticonvulsant, antipsychotics, antimigraine agent |
| CRPS 01 | opioid |
| CRPS 02 | opioid, antidepressant |
| CRPS 03 | opioid, anticonvulsant |
| CRPS 04 | opioid, anticonvulsant, antidepressant |
| CRPS 05 | opioid, anticonvulsant, benzodiazepine |
| CRPS 06 | anticonvulsant, antidepressant, benzodiazepine |
| CRPS 07 | opioid, antidepressant, benzodiazepine |
| CRPS 08 | anticonvulsant, antidepressant, benzodiazepine, anxiolytics |
| CRPS 09 | anticonvulsant, antidepressant, benzodiazepine |
| CRPS 10 | No medication |
| CRPS 11 | opioid, anticonvulsant, antidepressant, benzodiazepine |

CNS: central nervous system, FM: fibromyalgia, CRPS: complex regional pain syndrome
